# Recent Advances in Simultaneous Desulfurization and Denitrogenation of Fuel Oil

**DOI:** 10.3390/molecules31020279

**Published:** 2026-01-13

**Authors:** Jianrui Wang, Rui Wang

**Affiliations:** School of Environmental Science and Engineering, Shandong University, Qingdao 266237, China

**Keywords:** simultaneous desulfurization-denitrogenation, hydrogenation, fuel oil, extraction, adsorption, catalyst

## Abstract

The elimination of nitrogen and sulfur compounds from liquid fuel is a critical aspect of reducing environmental pollution. However, the widely utilized hydrodesulfurization and hydrodenitrogenation technologies require harsh operating conditions. Moreover, when operated simultaneously, these processes induce mutual competition and inhibition between the two reactions, thereby limiting the actual removal efficiency. Conversely, non-hydrogenation technologies offer substantial advantages in terms of operating conditions and provide high levels of desulfurization and denitrogenation. Nevertheless, the presence of nitrogen-containing compounds has also been demonstrated to engender competition and inhibition. It is imperative to develop environmentally friendly technologies that can simultaneously desulfurize and denitrogenate. This paper reviews research progress in this field over the past decade, providing a detailed assessment and comparison of hydrogenation and non-hydrogenation technologies, including adsorption, extraction, oxidation and biological methods. Furthermore, it considers future research directions. The article’s aim is to furnish a novel perspective on the development of clean fuel sources and to investigate more economical, sustainable, and commercially viable desulfurization and denitrogenation methods.

## 1. Introduction

Energy and environmental issues have long been recognized as a major stumbling block to social development [1]. The continuous increase in energy consumption has led to the depletion of low-sulfur and low-nitrogen crude oil, resulting in increasingly serious air pollution. Concurrently, unconventional fuel resources such as oil shale, oil sands and coal have been exploited to obtain high-sulfur crude oil [2]. The sulfur and nitrogen-containing compounds (SCCs and NCCs) found in liquid fuels are the primary cause of air pollution and extreme weather conditions, including haze and acid rain [3]. This phenomenon gives rise to a series of issues, including, but not limited to, soil acidification and building corrosion. The presence of SOx in the atmosphere has been demonstrated to combine with other pollutants to form carcinogens such as PM2.5, which have been shown to cause severe damage to the human respiratory system [4,5]. Concurrently, NO_x_ undergoes a chemical reaction with VOCs under ultraviolet radiation to generate photochemical smog, which has been demonstrated to irritate the skin and respiratory system. The organic nitrogen present in fuel displays strong oxidizing activity. While in storage, it is susceptible to a reaction with oxygen, resulting in the formation of gum and sediment. In fuel vehicle systems, the resulting sulfur oxides and nitrogen oxides are absorbed by the engine oil, accelerating its ageing and causing corrosion of the engine and exhaust system [6]. In addition, diesel engines produce more nitrogen oxides [7]. The six pollutants announced by the EPA include particulate matter, ozone, carbon monoxide, sulfur oxides, nitrogen oxides and lead. In the Tier 3 standards implemented by EPA, the NO_x_ limit is further reduced to 0.01 g/mile, and it is expected that in the new regulations in 2027, the NO_x_ limit will be further reduced to 0.02 g/bhp hr. The minimum requirement for SO_2_ emissions from factories is an emission limit of 100 mg/m^3^. In 2021, the US Environmental Protection Agency also recognized the close connection between sulfur and nitrogen compounds in air pollution and atmospheric chemistry, combining the study of the two substances.

Fuel is inextricably linked to daily life and industrial development. With clean energy and renewable resources impacting the traditional fuel sector and increasingly strict standards being set for the sulfur and nitrogen content of fuel, in order to reduce extreme weather conditions, it is essential to utilize advanced scientific and technological methodologies in order to control air pollution at its source, such as photochemical pollution and acid rain [8,9]. Currently, most literature covers studies on fuel desulfurization [10,11,12,13,14] and denitrogenation [15,16], but reports on simultaneous desulfurization and denitrogenation are much rarer [17]. Although several review articles have examined various aspects of the simultaneous desulfurization and denitrogenation of fuels, most have focused on specific fields or technologies. There has been no systematic and comprehensive work to integrate the reaction mechanisms, catalytic materials and key influencing parameters of all major methods into a unified framework, with a lack of systematic summaries and comprehensive comparisons. In consideration of this restriction, this review comprehensively discusses the hydroprocessing processes and underlying mechanisms of major nitrogenous and sulfur-containing compounds in liquid fuels, while presenting an in-depth analysis of simultaneous hydrodesulfurization and hydrodenitrogenation (HDS/HDN) technologies. Subsequently, it focuses on the fundamental principles and technical details (including reaction mechanisms) of four emerging non-hydrogenation approaches—simultaneous adsorptive desulfurization and denitrogenation (ADS/ADN), simultaneous extractive desulfurization and denitrogenation (EDS/EDN), simultaneous oxidative desulfurization and denitrogenation (ODS/ODN), simultaneous biological desulfurization and denitrogenation (BDS/BDN)—and systematically evaluates the advantages and limitations of each technology for concurrent fuel desulfurization and denitrogenation. Overall, this review aims to address the existing fundamental gap by providing a comprehensive. The aim of this review is to address the existing gap in the field by offering a comprehensive comparison and specific analysis of traditional hydrodesulfurization and denitrogenation and emerging non-hydrotreatment technologies. It also provides valuable discussions and promotes the development of clean fuel.

## 2. Typical Sulfur and Nitrogen Compounds in Fuel Oil

There is significant competition and inhibition between traditional hydrodesulfurization and hydrodenitrogenation. The specific mechanisms will be explained in detail in the following sections. Therefore, a full understanding of the different types of sulfur- and nitrogen-containing compounds in fuel is necessary.

### 2.1. Types of Sulfur Compounds

**Figure 1:**
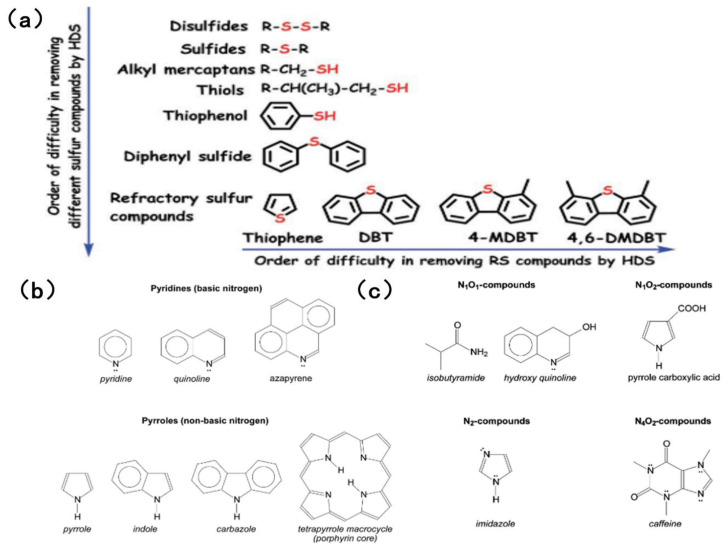
(**a**) Order of organic sulfur compounds in the HDS process based on removal difficulty in fuel feedstock. Reproduced with permission from Ref. [20]. Copyright 2020 RSC. (**b**) Examples of pyridinic and pyrrolic compounds. Reproduced with permission from Ref. [6]. Copyright 2017 ACS. (**c**) Examples of nitrogen-containing compounds with more than one heteroatom that were identified in crude oil samples. Reproduced with permission from Ref. [6]. Copyright 2017 ACS.

The sulfur content of fuel oil varies depending on the storage layer and type of petroleum, ranging from 0.05% to 14% [18,19]. Sulfur compounds can be classified as acidic or non-acidic based on their acidity or alkalinity. Simple sulfides, such as disulfide and hydrogen sulfide, can be easily removed by hydrogenation. However, aromatic sulfides with complex structures, such as thiophenes, benzothiophenes and dibenzothiophenes, pose a greater challenge in terms of their elimination, owing to resonance stability and spatial site resistance effects. Figure 1a shows common sulfides in HDS and their order of difficulty in removal [20]. When simulating sulfur compounds in fuel, BT, DBT and 4,6-DMDBT are most commonly chosen as the simulated sulfur compounds. This is because traditional HDS is relatively inefficient at removing aromatic sulfides.

### 2.2. Types of Nitrogen Compounds

Compared with the sulfur content, the nitrogen content of fuel is generally lower [21]. The nitrogen oxides in fuel are typically organic nitrogen, accounting for over 95% of the total, with only trace amounts of inorganic nitrogen present. Fuel is categorized as high-nitrogen (≥0.25 wt%) or low-nitrogen (<0.25 wt%) according to its nitrogen content. The fuel’s boiling point also varies with the concentration of nitrogen-containing compounds [6]. Organic nitrogen can be further categorized as either acyclic or aromatic compounds. The former are mainly organic amines, which have a low content and are easy to remove. The latter can be further classified as either alkaline (pyridine derivatives) or non-alkaline (pyrrole derivatives). An excessively high content of aromatic hydrocarbons reduces the quality of the fuel [22]. In most fuels, the ratio of alkaline to non-alkaline nitrogen fluctuates within the range 0.25–0.35 [23,24]. It is evident that basic nitrogen, a six-ring pyridine compound, which includes pyridine and quinoline, primarily exists in light fuel oil. These compounds are highly alkaline and interact strongly with acidic sites on the catalyst surface. Non-basic nitrogen, namely pentacyclic pyrrole compounds, mainly exist in heavy fuel and mainly include pyrrole, indole and carbazole, which makes the deep desulfurization of fuel difficult [25].

Although there are many types of nitrogen-containing substances, references [21,25] generally agree that the most abundant in crude oil are heterocyclic compounds containing a single nitrogen atom, such as pyridine, quinoline and carbazole. Despite the fact that the content of nitrogen compounds is much lower than that of sulfur compounds, the toxicity of nitrogen compounds to the catalyst is significant in the traditional hydrodesulfurization and hydrodenitrogenation process, forcing the catalyst to lose its desulfurization activity [21]. Figure 1b [6] shows common nitrogen-containing compounds in fuel.

## 3. Hydrodesulfurization and Hydrodenitrogenation

The HDS and HDN methodologies have been extensively applied in actual industrial production contexts. In the presence of elevated temperatures and pressures, in a hydrogen atmosphere, and with the aid of catalysts, the C-S and C-N bonds in sulfur and nitrogen compounds, respectively, are broken and subsequently converted into hydrogen sulfide (H_2_S) and ammonia (NH_3_) for removal.

### 3.1. Hydrodesulfurization (HDS)

Hydrodesulfurization is typically performed under harsh conditions (a temperature of 300–400 °C and a pressure of 3–10 MPa) using hydrogenation to break the C–S bond and release H_2_S and sulfur-free hydrocarbons [26]. This process occurs through two main reaction mechanisms: In the direct mechanism, the C-S bond is broken and replaced with hydrogen through hydrogenolysis or direct desulfurization (DDS) [27]. Aromatic sulfur compounds with linear structures, such as sulfides and mercaptans, are prone to reacting and can be removed by DDS. The other mechanism is indirect, also known as the hydrogenation pathway (HYD). This involves hydrogenating the sulfur compounds to generate corresponding saturated intermediate products, thereby achieving cleavage of the C-S bonds and desulfurization.

**Figure 2:**
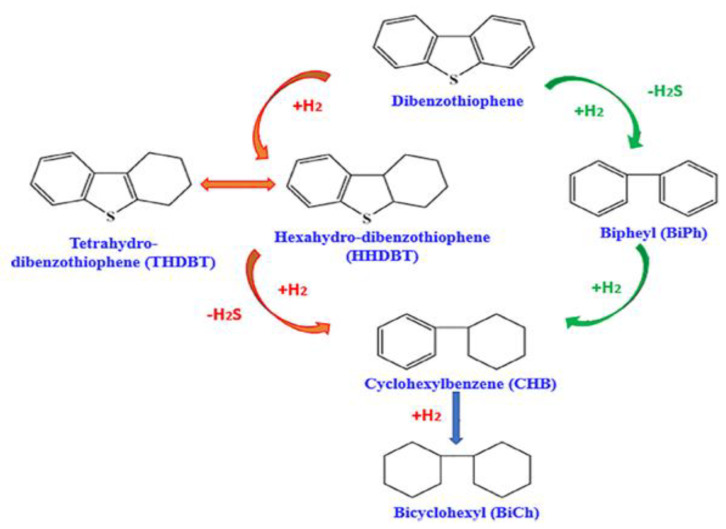
Possible pathways of HDS of DBT compound, reproduced with permission from Ref. [29]. Copyright 2018 Elsevier.

Dibenzothiophene (DBT) and its derivatives are the most difficult sulfur compounds to remove. They are mainly produced through the HYD method, which involves hydrogenation to form saturated aromatic rings, followed by C-S bond cleavage [28]. The specific reaction is shown in Figure 2 [29]. During the actual desulfurization reaction process, many sulfides react through different pathways during hydrodesulfurization (HDS). The research results of Sharifvaghefi et al. [30] indicate this. First, DBT undergoes hydrogenolysis, which directly breaks the C-S bond to release H_2_S; the final product is biphenyl. Secondly, via the hydrogenation pathway, DBT is first converted to tetrahydrodibenzothiophene (THDBT). The two pathways depend on the type of catalytic material, promoter, and support material used. Zheng et al. [31] reported that, in the thiophene HDS process, the final products of the DDS and HYD routes were all four compounds.

### 3.2. Hydrodenitrogenation (HDN)

The process of hydrodenitrogenation has been demonstrated to achieve ring-opening denitrogenation through the hydrogenation of aromatic rings. Subsequent to the cleavage of the C-N bond, alkenes, ammonia and various nitrogen compounds are released. The various reaction mechanisms in hydrodenitrogenation (HDN) are primarily contingent on the molecular structure, basicity, and the interaction mode with the active site of the catalyst. Prior to the cleavage of the C-N bond, there are substantial disparities in the structural characteristics of the five-membered N-ring and six-membered N-ring amine intermediates formed during the reaction [32]. In the context of polycyclic nitrogen compounds, two predominant HDN mechanisms have been identified: aniline-like and pyridine-like. The aniline-like mechanism focuses on the hydrogenation of the benzene ring and the cleavage of the C-N bond [33], while the pyridine-like mechanism involves the hydrogenation saturation of the nitrogen heterocyclic ring and the cleavage of the C-N bond [34]. It has been established that the remaining nitrogen compounds possess a single reaction mechanism. In comparison with non-alkaline compounds, nitrogen compounds with strong alkalinity exhibit enhanced adsorption properties on the active sites of the catalyst. This phenomenon has been observed to induce catalyst poisoning and result in competition with sulfur compounds for the active sites. It has a significant impact on the reaction process of HDN and HDS [35,36]. Furthermore, the low resolution of nitrogen compounds at the active sites results in irreversible poisoning [37].

**Figure 3:**
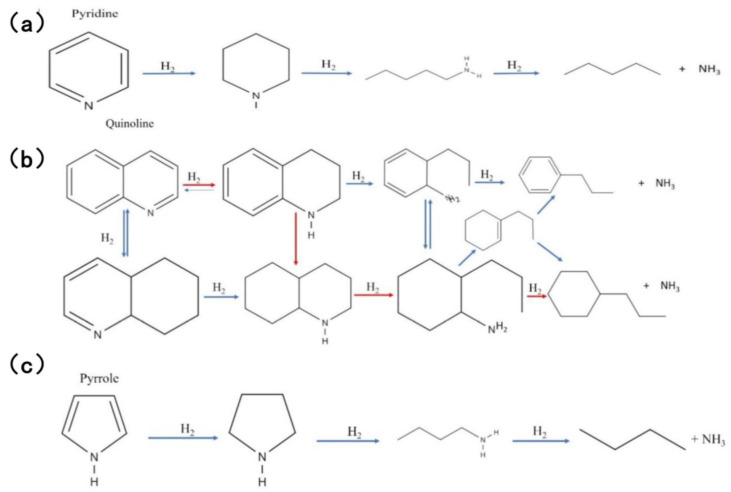
Reaction HDN network for basic N-compounds: (**a**) pyridine, (**b**) quinoline, (**c**) pyrrole. Reproduced with permission from Ref. [26]. Copyright 2021 ACS.

The reaction process of pyridine HDN, the simplest aromatic compound in fuel oil, is shown in Figure 3a. The process of hydrogenation of pyridine is gradual, resulting in the formation of piperidine and Amylamine. Subsequent removal of NH3 leads to the final formation of a C_5_ hydrocarbon [38,39,40]. Quinoline, a nitrogen compound with a high molecular weight and a boiling point above 340 °C, has been found to possess two HDN pathways [39,41,42]. The initial pathway entails hydrogenation, the process of saturating the ring, with the objective of synthesizing 1,2,3,4-tetrahydroquinoline (THQ). Subsequent to this, ring cleavage is initiated, resulting in the formation of orthopropylaniline (OPA). The final step in this process involves denitrogenation. The second pathway involves the saturation of THQ to decahydroquinoline (DHQ), followed by the opening of the ring to release hydrocarbons through PCHA. The specific reaction process is illustrated in Figure 3b [26]. In contrast, the reaction mechanism of pyrrole is relatively straightforward. Moravek et al. proposed a reaction process that is analogous to that depicted in Figure 3c.

### 3.3. Simultaneous Hydrodesulfurization and Hydrodenitrogenation

**Figure 4:**
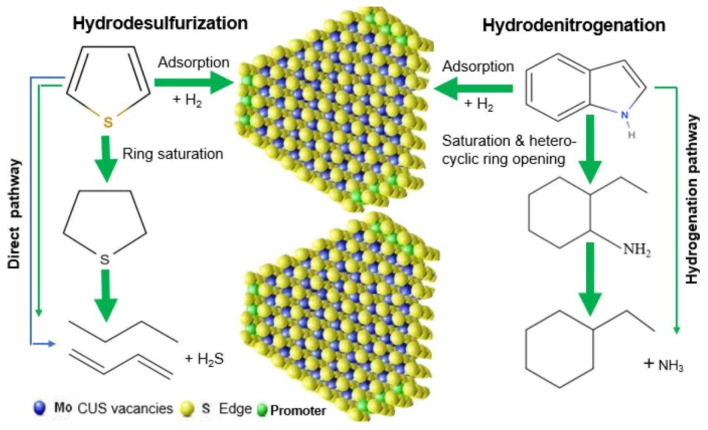
Catalysis of HDS and HDN for sulfur and nitrogen removal. Reproduced with permission from Ref. [26]. Copyright 2021 ACS.

In the process of actual fuel purification, hydrodesulfurization (HDS) and hydrodenitrogenation (HDN) occur in a concurrent manner. The most prevalent catalyst systems for this purpose involve loading Al_2_O_3_ with sulfide cobalt–molybdenum (Co-Mo) or nickel–molybdenum (Ni-Mo), with the coordination unsaturated sites (CUS) constituting the fundamental element of the catalyst activity for HDS and HDN. The formation of this compound is primarily attributable to the exposure of coordinated unsaturated molybdenum (or tungsten) atoms at the edges of MoS_2_ or WS_2_ crystals [43]. These sites possess Lewis acidity and have been demonstrated to adsorb and activate sulfur and nitrogen compounds, thereby promoting the cleavage of C-S and C-N bonds [44]. The specific catalytic pathways are illustrated in Figure 4.

As previously stated, the competition of nitrogen compounds for the active site, in conjunction with the irreversible poisoning of the catalyst, significantly impedes the HDS reaction. Therefore, it is essential to comprehend the interplay between these two reactions and the selection of the active site of the catalyst. When De Mello et al. [45] simultaneously carried out HDS and HDN using the NiMoP/Al_2_O_3_ catalyst, a similar result was discovered: the competition of active sites led to a decrease in HDS efficiency. Specifically, the conversion rate of 4-6-DMDBT decreased from 85% for individual conversion to 65%, and the conversion rate of Q decreased from 80% to 75%. In subsequent studies, Albersberger et al. [46] discovered that when DBT and o-propyl aniline were HDS and HDN simultaneously, they would share the same active site. Due to the preferential adsorption of o-propyl aniline by the active site, the HDS reaction process was greatly inhibited. The conversion rate of DBT decreased by 31% compared to individual conversion, while the conversion rate of Q or OPA only decreased by about 6% during competition. The research of Farag et al. [47] indicates that the presence of quinoline in HDS and HDN without MoS_2_ has an adverse effect on the HDS of DBT, but the presence of DBT promotes the HDN process of quinoline by increasing the acidity of potential active sites. Similarly, under competitive reactions, the conversion rate of DBT decreased by 31%, while the conversion rate of Q or OPA only decreased by 4% during competition.

In order to surmount the competitive and inhibitory relationship between HDS and HDN, the design of hydrogenation treatment catalysts with higher activity than traditional catalysts has always been a significant issue. Oliver et al. [48] sought to explore different catalyst carriers of (nickel) molybdenum sulfide catalysts with a view to improving catalytic performance. The research findings indicate that the characteristics of the carrier have a significant impact on the dispersion of the unpromoted catalyst and the degree of modification of nickel at the periphery of the Ni-Mo-S phase. In addition, the acidity of the carrier has been observed to influence the acidity of the loaded sulfide phase, a factor that may play a crucial role in HDN. The catalyst designed in this experiment, in the system with the best reaction efficiency, showed a 17% decrease in the conversion rate of sulfides and a 3% decrease in the conversion rate of nitrides even under competitive conditions, which is much higher than the previous research results. In their study, Idia et al. [49] investigated the kinetic modelling of the hydrodesulfurization (HDS) reactions of 4,6-dimethyldibenzothiophene (4,6-DMDBT) and the hydrodenitrogenation (HDN) reactions of quinoline with CoMoP and NiMoP catalysts in a stacked bed configuration. The research results show that a higher reaction temperature can not only increase the total conversion rate of 4,6-DMDBT and quinoline, but also enhance the hydrogenation selectivity. Even during competitive conversion, the CoMoP and NiMoP stacking configuration at 340 °C can achieve a conversion rate of 85% for 4,6-DMDBT and 95% for Q. Matheus et al. [45] conducted a study on the competitive effect of phosphorus content on hydrodesulfurization (HDS) of 4,6-dimethyldibenzothiophene (4,6-DMDBT) and hydrodenitrogenation (HDN) of quinoline. It has been demonstrated that when the content of quinoline is relatively high, the HDS activity of all catalysts is inhibited. Among these catalysts, the inhibition of the hydrogenation (HYD) pathway is more obvious. In instances where the concentration of quinoline is minimal, the addition of phosphorus has been observed to augment the activity of HDS. For specific quinoline concentrations and temperatures, the catalyst containing 1 wt% phosphorus is more active for HDS, while the catalyst containing 2 wt% phosphorus has a stronger inhibitory effect on HDS but is active for HDN.

In conclusion, although hydrogenation is widely used in actual production, to deal with fuel with high sulfur and nitrogen content, not only is an additional 50% or more of hydrogen required, but also several times the amount of catalyst is needed. Concurrently, the HDN and HDS reactions characteristically exhibit a robust interaction in hydrogenation treatment. By summarizing the literature, it is found that in hydrodesulfurization and hydrodenitrogenation, the inhibition effect of nitrides on sulfides is much higher than that of sulfides on nitrides. The core reason is that the adsorption affinity and competitive priority of nitrides on catalytically active sites are significantly higher than those of sulfides. Specifically, nitrogen atoms containing strong alkaline lone pair electrons in nitrides can form a strong σ-bond adsorption with Lewis acid sites on the catalyst surface. Sulfides are adsorbed by sulfur atoms or π electrons in aromatic rings, and the adsorption bond energy is only 1/3–1/2 of that of nitrides. The removal of sulfide is more dependent on the HYD path, while nitrogen compounds just strongly inhibit the HYD path, forcing sulfide to fail to complete aromatic hydrogenation, resulting in a sudden decline in desulfurization efficiency. A comprehensive understanding of the mechanisms of active site competition, reaction pathway differences, and product inhibition, in conjunction with the meticulous design of catalysts, has enabled the development of novel catalysts that exhibit high activity and selectivity. These catalysts are well-suited to meet the progressively stringent requirements for fuel desulfurization and denitrogenation.

## 4. Non-Hydrogenation Process

The conventional hydrodesulfurization and hydrodenitrogenation (HDS/HDN) processes necessitate the use of hydrogen, a costly reactant, as a fundamental component of the reaction. These processes are characterized by their relatively low overall efficiency in the production of ultra-clean fuel and their inability to adequately remove refractory aromatic compounds. Consequently, researchers have developed a range of non-hydrogenation technologies, including oxidative denitrogenation (ODN)/oxidative desulfurization (ODS), adsorption denitrogenation (ADN)/adsorption desulfurization (ADS), extractive denitrogenation (EDN)/extractive desulfurization (EDS), and biological denitrogenation (BDN)/biological desulfurization (BDS). These have been complemented by photocatalytic and ultrasonic-assisted oxidative desulfurization and denitrogenation technologies.

### 4.1. Simultaneous Extraction Desulfurization and Denitrogenation

Extraction is a process that involves the removal of sulfur and nitrogen compounds from fuel under mild conditions. It achieves this by selectively dissolving these heteroatom compounds, thereby separating them from the fuel components. The core of this methodology is the selection of an efficient and highly selective extractant. In the present study, ionic liquids (ILs) and deep eutectic solvents (DESs) are regarded as highly promising extractants due to their unique properties.

#### 4.1.1. Ionic Liquids (ILs)

**Figure 5:**
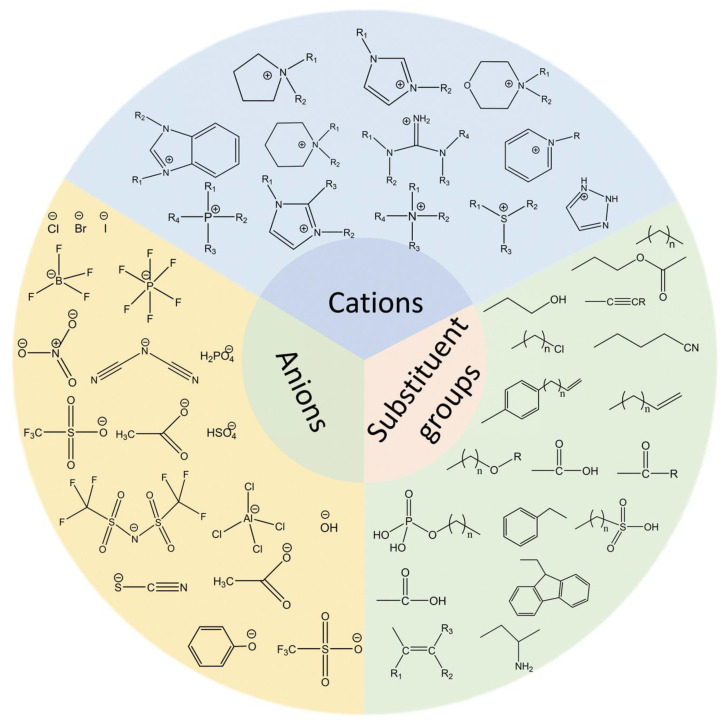
Structures of the typical IL cations, anions, and substituent groups. Reproduced with permission from Ref. [56]. Copyright 2023 RSC.

Ionic liquids have been shown to exhibit a series of advantageous physical and chemical properties when compared with traditional aprotic solvents. This renders them an optimal choice for a range of applications, including extraction, desulfurization and denitrogenation [35,50,51]. The extraction and separation of aromatic compounds from any fuel source is possible, thereby ensuring higher extraction yields. Furthermore, these substances exhibit minimal vapor pressure and remarkably low evaporation during the extraction process, thus classifying them as a novel type of green solvent. The substance under scrutiny is chiefly constituted of molten salts of organic cations with deficient coordination and inorganic or organic anions [52,53]. It is noteworthy that even the most minimal alteration in its structural configuration has the capacity to induce significant alterations in its physical and chemical properties [54]. In practical applications, the selectivity, extraction capacity, surface activity and other properties of ionic liquids as extractants must be considered [55]. The properties of these liquids are determined by the structure of the ionic liquids themselves, as well as the different anions and cations that are present. Common anions, cations and substituents are illustrated in Figure 5 [56].

The mechanism of simultaneous desulfurization and denitrogenation by ionic liquids is intricate and typically involves multiple synergistic effects. The primary mechanisms encompass acid–base interactions, hydrogen bond interactions, π–π interactions, Lewis acid–base interactions, and others. Yi Nie et al. [57] were responsible for the design and synthesis of three types of bifunctional ionic liquids for the purpose of simultaneous desulfurization and denitrogenation. The findings indicate that the three nitrogen-containing compounds (pyridine, pyrrole, and quinoline) can be effectively removed, with a removal rate approaching 100%. It has been demonstrated that quinoline exerts no influence on the ultimate DBT removal; its effect is merely negligible in the preliminary phase of the reaction. It has been demonstrated that both pyridine and pyrrole have a deleterious effect on desulfurization. Of these, pyrrole has been shown to be the most significant inhibitor. This phenomenon is primarily attributable to the oxidative competition between nitrogen-containing compounds and sulfides, as well as the interference with the extraction capacity of ionic liquids. Compared with the traditional Sulfolane and conventional imidazole ionic liquids, the extraction effect of bifunctional ionic liquids is remarkable. After 4 times of reuse, the DBT removal rate was still 91.2%, the rate constant decreased from 0.9323 min^−1^ to 0.9141 min^−1^, and the activity decreased by only 2%. In their seminal work, Hansmeier et al. [58] developed an ionic liquid with a specific structure (pyridinium group containing ${N(CN)}_{2}^{-}{,SCN}^{-}$ anions) as an efficient extractant for fuel desulfurization and denitrogenation. The performance of the developed ionic liquid was found to be significantly superior to that of the industrial standard sulfoxide. The extraction selectivity of ionic liquids for nitrogen compounds is significantly higher than that for sulfur compounds. Compared with the reference solution, the extraction efficiency of the four designed extractants for nitrides and sulfides was increased by more than 10%, the extraction efficiency of nitrides was up to 99%, and the extraction efficiency of sulfides was higher than 84%. After repeated use for 5–8 times, the single extraction rate of sulfide remained above 75%, while the extraction rate of nitride remained above 95%. Chen et al. [59] selected four acidic ionic liquids (two Lewis acidic and two Brown acidic) for simultaneous desulfurization and denitrogenation. The findings demonstrate that the Lewis acid type exhibits superior performance. The extraction efficiency of this acidic ionic liquid is much higher than that of the traditional solvent. The desulfurization rate is up to 95.9%, and the denitrogenation rate is nearly 100%. It can be reused five times and still maintain stable activity and the regeneration method is simple. These are primarily attributable to the coordination of cations with sulfur and nitrogen atoms, and the synergistic acidic effect of anions. Furthermore, the study revealed that acidic ionic liquids exhibited remarkably high selectivity for the removal of basic nitrogen compounds (e.g., pyridine), thus providing a viable solution to the poisoning problem of HDS catalysts.

#### 4.1.2. Deep Eutectic Solvents (DESs)

Deep eutectic solvents are typically constituted of two components: hydrogen bond acceptors and hydrogen bond donors. These are low-eutectic mixtures formed under the interaction of hydrogen bonds [60,61]. DES shares numerous similarities with ILs, including low vapor pressure, wide liquid range, high thermal stability, low flammability and high solubility [62]. However, DESs have a low preparation cost, a simple preparation process, better economic appeal, and are biodegradable with low toxicity. It is also a green solvent. As demonstrated in earlier research, the extractant in question has been shown to have the capacity to remove sulfur oxides (EDS) [63,64] and nitrogen oxides (EDN) [65] in a simultaneous manner [66]. Its remarkable performance in simultaneous desulfurization and denitrogenation has led to a surge in research interest in DESs.

**Figure 6:**
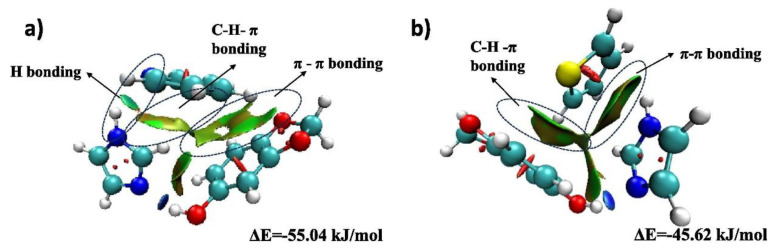
Optimized conformations for SES: IMI-1 with (**a**) pyridine (**b**) thiophene. Reproduced with permission from Ref. [69]. Copyright 2025 Elsevier.

Lima et al. [67] made a significant contribution to the field by achieving research progress in the simultaneous EDS and EDN. The efficacy of four types of DES was investigated through the preparation of these substances and the subsequent simulation of the fuel oil and gasoline systems. The findings of this investigation revealed that the four types of DES exhibited an efficiency range of 65% to 99% in their capacity to target nitrogen and sulfur compounds. Sulfur compounds were the most challenging to eliminate, with Th exhibiting the most significant difficulty and EE demonstrating the least. The removal efficiency of nitrogen compounds is higher (Py can reach over 99%). The findings demonstrate that DES exhibits notable stability in the removal of nitrogen compounds, unaffected by the complexity of the fuel matrix. The deep removal of sulfur compounds can be achieved through multi-stage extraction. The extractant can be completely recovered by precipitation with water, vacuum drying and regeneration, and can be recycled for more than five times. This conclusion was corroborated by Rogosic et al. [68]. In the course of the experiment, ChCl was utilized as the hydrogen bond acceptor, while PG was employed as the hydrogen bond donor. This approach enabled the preparation of DES, which exhibited a molar ratio of 1:3 for desulfurization and denitrogenation. Compared with sulfolane, the selectivity of Des for nitrides was significantly improved, but the selectivity for sulfides was only 30–40%. MK et al. [69] designed three DES with imidazole as the hydrogen bond acceptor and natural compounds as the hydrogen bond donor for the purpose of removing sulfur and nitrogen from the fuel. The high polarity of DES is attributable to the strong hydrogen bond formed between the N atom of imidazole and pyridine, and the π–π interaction between the aromatic rings of imidazole and HBD and thiophene (Figure 6). The enhanced selectivity for polar impurities (pyridine, thiophene) is a consequence of these interactions. The synergistic effect of multiple mechanisms enables all three DES to efficiently and concurrently desulfurize and denitrify, although the denitrogenation effect is significantly superior to that of desulfurization. In the study by Zarin et al. [70], the authors prepared acidic DES utilizing triphenylphosphine bromide as the hydrogen bond acceptor and p-aminobenzoic acid as the hydrogen bond donor, with a molar ratio of 2:3. The removal performance of thiophene and pyridine in synthetic gasoline was systematically evaluated. The experimental results show that the removal efficiency of pyridine is 50% higher than that of thiophene, and the RMSD of extractant is less than 0.005, which is higher than that of conventional solvents. Furthermore, the efficacy of the device in removing impurities from real gasoline is significantly superior to that of analogous products. It is widely acknowledged that the efficacy of DES as a denitrifying agent is contingent upon its capacity to selectively extract nitrogen and sulfur compounds. This property is a consequence of the agent’s unique extraction mechanisms, which are distinct for both nitrogen and sulfur compounds.

#### 4.1.3. Other New Types of Extractants

**Figure 7:**
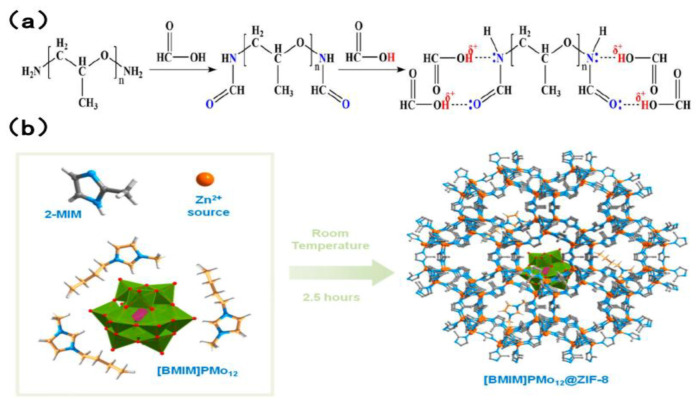
(**a**) Formation of PEA/FA. Reproduced with permission from Ref. [66]. Copyright 2020 ACS. (**b**) Representation of the preparation of the [BMIM]PMo12@ZIF-8 compound by a “bottlearound-the ship” approach and using a sustainable room temperature synthetic strategy (one-pot and in situ). Reproduced with permission from Ref. [71]. Copyright 2020 MDPI.

In the context of industrial extraction, desulfurization, and denitrogenation, both ILS and DESs have been shown to exhibit certain undesirable characteristics. The use of EDS extractant, for instance, has been associated with significant environmental damage, cumbersome recovery processes, and considerable expense. Conversely, EDN necessitates the simultaneous removal of both alkaline and non-alkaline nitrogen, a task that is rarely accomplished by available extractants. Consequently, there is a pressing need to develop an extractant that is both cost-effective and highly efficient, with the capacity to facilitate desulfurization and denitrogenation processes. Zhu et al. [66] made a significant contribution to this aspect of research. A novel composite solvent was developed, comprising a new type of polyetheramine (PEA/formic acid FA complex). It has been established that PEA reacts with FA to form amide groups. Concurrently, a complexation effect is observed between excess FA and amide groups. The complex is characterized by the presence of both sufficient Lewis basicity and mild Brownst acidity. The interaction between sulfur and nitrogen compounds is primarily characterized by weak hydrogen bonds and mild acid–base interactions. The extractant, with its dual Lewis alkalinity and mild Brunst acidity, enables efficient removal of sulfur and nitrogen compounds from fuel. Silva et al. [71] encapsulated imidazole-based polyoxometriate ([BMIM]_3_PMo_12_) into the ZIF-8 molecular sieve framework to prepare the [BMIM]PMo_12_@ZIF-8 heterogeneous catalyst, whose structure is shown in Figure 7. The catalyst can be repeatedly recycled up to ten times. The desulfurization efficiency has been found to be consistently above 98.5%, while the denitrogenation efficiency has been found to be stable at approximately 82%. The apparatus is capable of achieving efficient simultaneous desulfurization and denitrogenation, and complete removal of the target impurities in the fuel within a time frame of one hour.

In conclusion, the extraction and removal of sulfur and nitrogen compounds is a very promising method that can efficiently remove most of the pollutants and impurities in a short period of time. In comparison with aprotic solvents, both ILS and DESs have been demonstrated to exhibit significant superiority, thus providing valuable references for future research and application [72]. However, the nitrogen removal efficiency of these two extractants is significantly higher than that of desulfurization. This significant disadvantage makes the extraction method need to be used many times in desulfurization, which undoubtedly increases the cost.

### 4.2. Simultaneous Adsorptive Desulfurization-Denitrogenation

Adsorption desulfurization and adsorption denitrogenation (ADS/ADN) are comparable to extraction processes, and the selection of an appropriate adsorbent is pivotal to achieving optimal results. The principal requirements for adsorbents encompass operational conditions, methodologies for preparation, elevated levels of porosity, and environmental sustainability. The most commonly used adsorbents include activated carbon (ACs), mesoporous silica, metal oxides (Al_2_O_3_, TiO_2_, ZnO), metal–organic frameworks (MOFs), and zeolites [73]. The subsequent section will provide a comprehensive discussion of several significant adsorbents, a comparative analysis of the distinctions between various systems, and an in-depth examination of the subject.

#### 4.2.1. Metal-Supported Catalyst

Common metal-supported catalysts comprise metals with denitrogenation and desulfurization activities (e.g., Pt, Cu) that are loaded onto high specific surface area carriers (e.g., activated carbon, zeolite, alumina). It is evident that there is a high degree of similarity between the adsorption design concepts and mechanisms of the two systems. To illustrate this point, consider activated carbon (AC). It is a highly mature adsorbent in practical application, featuring a high specific surface area and different functional groups attached to its surface [74]. Typically, it is loaded with metal ions, such as Cu^+^ and Ni^2+^, or modified with acids and bases to enhance its adsorption capacity for sulfur/nitrogen compounds. The adsorption mechanism principally incorporates a series of intermolecular interactions, including π–π interaction, acid–base interaction, hydrogen bonding, and π complexation bonding. It is noteworthy that these mechanisms bear a striking resemblance to those involved in extraction.

**Figure 8:**
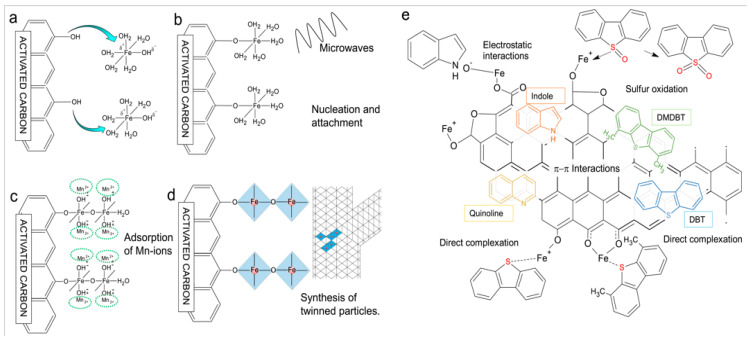
(**a**–**d**) Proposed steps for the formation of twinned particles in material F_4_-FeMn. Reproduced with permission from Ref. [75]. Copyright 2019 Elsevier. (**e**) Proposed adsorption mechanism of several molecules studied on the surface of the iron-modified activated carbon. DMDBT stands for 4,6-dimethyldibenzotiophene and DBT for dibenzothiophene. Reproduced with permission from Ref. [75]. Copyright 2019 Elsevier.

As demonstrated in Figure 8a–d, Arcibar et al. [75] concentrated on the development of iron-manganese microwave-modified activated carbon adsorbents for the purpose of achieving synchronous desulfurization and denitrogenation of fuel oil. It has been demonstrated that the synergistic mechanism of inducing the formation of twinked needle-like structures of iron oxides, the π–π interaction between the activated carbon matrix and aromatic impurities, the acid–base interaction between the acidic sites of iron oxides and nitrogen compounds, and the surface complexation between iron ions and sulfur atoms (Figure 8b), it was found that the comprehensive adsorption performance of F_4_-FeMn was the best. The maximum adsorption capacity for DMDBT is 0.391 mmol/g, and the adsorption capacities for indole and quinoline are 1.322 mmol/g and 0.756 mmol/L, respectively. Furthermore, it demonstrates a high degree of selectivity for the recalcitrant DMDBT. Arcibar et al. [76] modified activated carbon (AC-I) with iron nanoparticles with a view to enhancing the adsorption capacity of traditional nitrogen compounds. Concurrently, they investigated the influence of sulfur compounds on denitrogenation performance. The results demonstrate that the specific surface area of the modified material (AC-I) undergoes a slight increase, and the surface charge undergoes a transition from alkaline to weakly acidic. In the presence of both indole and quinoline, AC-I exhibited an indole adsorption capacity that was 32% higher than that of AC, while its quinoline adsorption capacity increased by 9%, indicative of its high selectivity for indole. In the presence of 50 ppm sulfur, the adsorption capacity of AC-I for indole and quinoline is still 20% and 8% higher than that of AC, respectively. However, the desulfurization capacity of AC-I is lower than that of AC. This indicates that iron modification enhances the selectivity of the material for nitrogen compounds, and the iron-nitrogen interaction is stronger than the iron-sulfur interaction. Thaligari et al. [77] developed a nickel-modified granular activated carbon (Ni-GAC) catalyst for the simultaneous desulfurization and denitrogenation of fuels. The successful loading of nickel results in the enhancement of the GAC surface uniformity, with the presence of nickel oxides. It is evident that the synchronous adsorption capacity of activated carbon for DBT and QN in liquid fuels was significantly enhanced by nickel modification. This phenomenon can be attributed to the acid–base interaction between Ni^2+^ and pollutants, as well as the hydrogen bond interaction of surface functional groups. Concurrently, experimental findings indicated that the adsorption capacity of QN with elevated alkalinity was superior to that of DBT, exhibiting a competitive dynamic between the two.

Numerous studies have modified carriers to enhance adsorption performance; however, in actual fuel, resins, aromatics, etc. compete with sulfur and nitrogen compounds for adsorption sites, resulting in limited adsorption capacity. The majority of modified activated carbons demonstrate high selectivity for nitrogen compounds; however, they frequently exhibit inadequate removal capacity for challenging pollutants such as 4,6-dimethyldibenzothiophene. In the context of industrial production, although the cycle times and specific thermal degradation points of adsorbents are obviously mentioned in the above three modified activated carbon experiments, according to the commonness of these adsorbents, the oxidation of activated carbon matrix will occur above 600 °C, and the regeneration method is single, which mainly depends on thermal regeneration, with high energy consumption and high cost, preventing its large-scale application.

#### 4.2.2. Metal–Organic Frameworks

**Figure 9:**
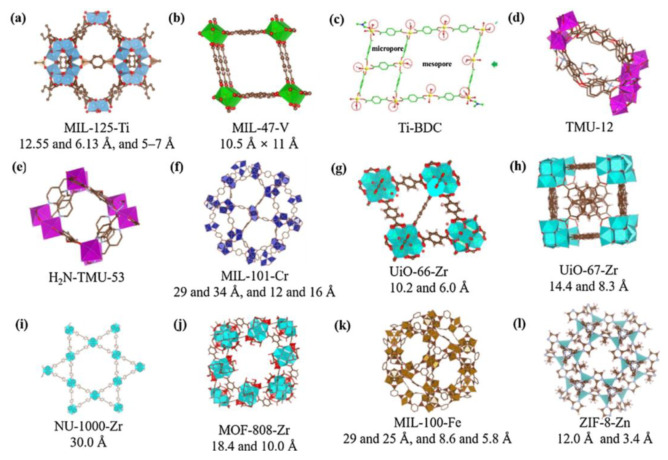
Structures of various MOFs. Reproduced with permission from Ref. [80]. Copyright 2019 Elsevier.

MOFs are defined as porous materials formed by the connection of inorganic metal ions or clusters with organic ligands through coordination bonds. The structural organization of these materials is such that they self-assemble into a three-dimensional network framework through coordination bonds between inorganic metal nodes (including single metal ions and multi-nuclear metal clusters) and organic ligands. This results in high porosity, high specific surface area and structural tunability [78,79]. The synthesis of diverse materials can be achieved through the alteration of metal ions or ligands, with these materials finding wide application in fields such as adsorption and degradation. As illustrated in Figure 9, a range of MOF structures is presented [80]. In comparison with inorganic zeolites, aluminium phosphate, and analogous structures, MOFs can be formed by different metals and a variety of organic ligands. These materials typically exhibit a highly porous structure, which is essential for their functionality in adsorption processes [81,82].

**Figure 10:**
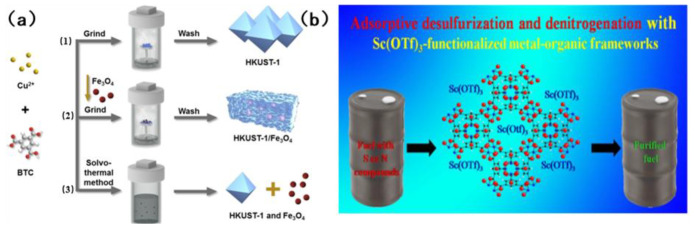
(**a**) Preparation method of HKUST-1/Fe_3_O_4_ composite material. Reproduced with permission from Ref. [83]. Copyright 2016 Elsevier. (**b**) The structure of Sc (OTF)_3_ adsorbent. Reproduced with permission from Ref. [84]. Copyright 2015 ACS.

Tan et al. [83] prepared HKUST-1/Fe_3_O_4_ composites using a novel dry gel transformation method (see Figure 10a). The composite in question is a magnetic MOF, which is characterized by the presence of both micropores and mesoporous pores. The open metal sites of quinoline and HKUST-1 are adsorbed through acid–base interaction. Indole, due to the presence of N-H bonds, has the capacity to form hydrogen bonds with the framework and thus ignores the effect of steric hindrance. This results in an adsorption capacity that is much higher than that of quinoline. However, the adsorption of sulfur compounds is contingent upon acid–base interaction and π complexation. The present study investigates the efficacy of an adsorption system, subject to an externally applied magnetic field, in the removal of aromatic sulfur and nitrogen compounds in fuel. The adsorption capacity of this adsorbent is superior to that of traditional adsorbents such as activated carbon and graphite. The adsorbent was separated by a magnetic field and regenerated. The recovery rate of the adsorbent was more than 99% each time. After six consecutive cycles, the cycle stability was outstanding. In the seminal study, Khan et al. [84] pioneered the loading of Sc(OTF)_3_ onto MOFs, marking a pivotal advancement in the field. Utilizing the inherent acidity of Sc(OTF)_3_, they demonstrated a remarkable enhancement in the adsorption selectivity for sulfur and nitrogen compounds through the judicious exploitation of acid–base interactions. Figure 10b illustrates the structure of the absorbent that undergoes modifications upon loading. The study found that the adsorption effects on benzothiophene, dibenzothiophene and quinoline were significantly increased by 64.7%, 20.7% and 34.2%, respectively. However, the absorption of indole did not show a significant increase. The underlying reason for this phenomenon is that it is incapable of forming an acid–base interaction with acidic Sc(OTF)_3_. Furthermore, following the loading process, the porosity of MOFs is reduced, thereby hindering the process of adsorption. The adsorbent can be stably recycled for 4 times, and the regeneration process is mild.

Metal–organic frameworks (MOFs) have demonstrated considerable potential in the domains of fuel desulfurization and denitrogenation, attributable to their elevated specific surface area and porous structural characteristics. In comparison with materials such as activated carbon and zeolite, the diversity of its preparation types and the variability of its structure greatly enhance the adsorption performance. However, when dealing with large-volume sulfur or nitrogen compounds that are difficult to remove, the clogging of pores leads to slow adsorption kinetics. Concurrently, the majority of MOF materials are confronted with defects such as competition for adsorption sites, elevated preparation costs, constrained thermal stability, and degradation of regeneration performance. These practical application problems bear a strong resemblance to those encountered in the field of activated carbon.

### 4.3. Simultaneous Oxidative Desulfurization-Denitrogenation

Oxidation desulfurization (ODS) and oxidation denitrogenation (ODN) in fuel are usually carried out in a system of oxidant and catalyst at normal temperature and pressure. Therefore, the selection of the catalyst and oxidant is a key link in the oxidation system. Sulfur oxides will be oxidized to the corresponding sulfoxides or sulfones [2], while the oxidation products of nitrogen oxides are more complex. Aniline is oxidized to azoxylbenzene, carbazole is oxidized to carbazole 1,4-dione, and the oxidation of indole produces polymer materials. In ODS/ODN, ozone and hydrogen peroxide are used as oxidants to destroy the C-S bond of organic sulfur compounds and functional groups of nitrogen-containing compounds in fuel oil through selective oxidation, and the low valent sulfur and nitrogen are oxidized into sulfur oxides such as sulfones and sulfonic acids and nitrogen oxides, carboxylic acids and other nitrogen-containing oxidation products, respectively. This oxidation method fundamentally changes the problem of the weak polarity of original sulfur compounds and nitrogen compounds and the small solubility difference with alkanes, and the generated oxides have stronger polarity and higher electronegativity, providing good conditions for subsequent separation. At the same time, the adsorbent/extractant used in the actual production can not only remove the nitrogen base, heavy metal ions and other components that may poison the oxidation catalyst in the real fuel, but also remove the oxidation products in time to reduce the probability of blocking the catalyst channel. Therefore, the efficient separation of oxidation products and fuel oil is often achieved by means of extraction, centrifugation and other separation methods, and ultimately, the purpose of deep purification of fuel oil is achieved. The oxidation method is always used in combination with extraction or adsorption [2], and can also be used in conjunction with auxiliary technologies such as visible or ultraviolet light irradiation and ultrasound [26] to enhance the removal rate. High selectivity catalysts are more convenient to control and improve removal efficiency (Table 1).

#### 4.3.1. Choice of Oxidant

In a reaction system where oxidation, desulfurization and denitrogenation are carried out simultaneously, the selection of the oxidant is of vital importance, which is usually dependent on the hydrophobicity of the catalyst. Oxidants, during the reaction process, release peroxide free radicals, provide reactive oxygen species to break the C-S and C-N bond of sulfur nitrogen compounds and oxidize them into polar and easily separable products. Consequently, the reaction rate of the oxidation process can be affected by the presence of inappropriate oxidants. As demonstrated in the extant research, the most prevalent oxidants encompass TBHP, hydrogen peroxide, molecular oxygen, O_3_, air, amongst others. O_3_ [85] has become an alternative to traditional catalysts due to its high selectivity, strong oxidizing properties, and environmental friendliness. It can oxidize pollutants in a short period of time by breaking the O-O bond. H_2_O_2_ is a widely used and environmentally friendly oxidant, with only H_2_O, but its biodegradability requires excessive addition to ensure sufficient active oxides. H_2_O_2_, as a byproduct, can be used as a co oxidant for heterogeneous thermal catalysts and also as the main oxidant in catalysis. TBHP, as an oxidant and heterogeneous catalyst in oxidation reactions, often leads to the formation of complexes and metal peroxide intermediates through nucleophilic attacks. The use of TBHP or H_2_O_2_ as an oxidant is usually in the form of an aqueous solution. Two phases are introduced into the reaction system to inhibit mass transfer and affect the reaction rate. Although molecular oxygen or air can overcome this obstacle when used as an oxidant, it requires high pressure and high purification, which greatly increases the cost [86]. It is evident that H_2_O_2_ demonstrates elevated reaction rates and selectivity under mild conditions, thereby exhibiting superior performance in comparison to other oxidants. It has been demonstrated in previous studies that, in the event of H_2_O_2_ being utilized as an oxidant, the presence of a hydrophobic catalyst is an essential requisite [87]. The efficacy of the process is optimized by the utilization of silicate catalysts, while metal oxide catalysts belonging to the IV-VI group demonstrate compatibility with tert-butyl hydrogen peroxide. Furthermore, as the temperature rises, H_2_O_2_ decomposes into O_2_ and H_2_O, thereby inhibiting the reaction. Excessive H_2_O_2_ has been shown to eliminate hydroxyl radicals with oxidizing effects, thereby seriously affecting the efficiency of desulfurization and denitrogenation [88]. Furthermore, the mechanism of oxidation reactions can also be subject to alteration depending on the different oxidants. When molecular oxygen is utilized as the oxidant, the predominant reaction mechanism is that of free radicals [89]. When H_2_O_2_ is utilized as the oxidant, the oxidation reaction progresses through the generation of metal peroxide complexes of the catalyst.

**Table 1:** Various Important Parameters of Catalysts in ODS.

Catalyst	n (Catalyst)	Oxidant	Reactant	O/S	Temperature (°C)	Time (min)	Conversion Rate	Ref.
[cetrimonium]_11_P_2_W_13_V_5_O_62_	7.5 g/L	H_2_O_2_	DBT; Quinoline	8	70	45	DBT:94% Q:100%	[90]
Tris-LDH-LaW_1_	N(DBT)/n(catalyst) = 20	H_2_O_2_	Sulfides: DBT, BT, 4,6-DMDBT; Nitrides: Quinoline, Pyridine, Indole	6	65	60	S:99%: N:99%	[91]
HPA-5	0.5 mmol	O_2_	Sulfides: (Benzothiophene, DBT 4,6-DMDBT); Nitrides (indole, 1-methylindole, 2-methylindole, 3-methylindole, quinoline, etc.); Quinalidine	—	120	—	N:100% (5 min); S:88% (24 h)	[92]
TA-700; AM-700	200 mg	H_2_O_2_	Sulfides (DBT, 4-methylDBT, 4,6-dimethylDBT, benzothiophene, thiophene), nitrides; SRGO; diesel	20	60	5	DBT:100% Diesel oil:S:97%;N:96%; SRGO:S:70%; N:89%	[93]
MoO_3_/Al_2_O_3_	5 wt%	CHP	Sulfides (4-methylDBT, 4,6-dimethylDBT) Nitrides, polycyclic aromatic hydrocarbons (PAHs); HGO1, HGO2	20	75	—	HGO_1_:S:83%; HGO_2_:S:39%	[94]
Mn−Co−Mo/Al_2_O_3_	0.1 g	H_2_O_2_ NaClO	Sulfides (DBT, BT, 4,6-DMDBT) Nitrides (pyridine, indole, carbazole)	4 O/N = 8	25	15	DBT:100% PY:100%	[95]
MAX-phase@rGO@PW_11_Zn	0.01 g	H_2_O_2_	Sulfides (thiophene, TH, thiols) Nitrides (pyridine, PY); gasoline	—	35	60	TH:97.72% PY:98.94%	[96]

The ‘—’ represents a value where the parameter does not appear.

As shown in Table 2, a detailed comparison of various commonly used oxidants was made from different perspectives. Combined with the research reports mentioned in the following section, in most cases, H_2_O_2_ and O_2_ are the better choices.

#### 4.3.2. Choice of Catalyst

Catalysts are the most crucial reactants in the oxidation system. In contrast to the complexity and inconvenience of the homogeneous catalytic system, the heterogeneous catalytic system is more favored. It has advantages such as easier separation, regeneration, and reusability of the catalyst. Nevertheless, regardless of the reaction system type, the time consumed for the reaction is extremely short.

**Figure 11:**
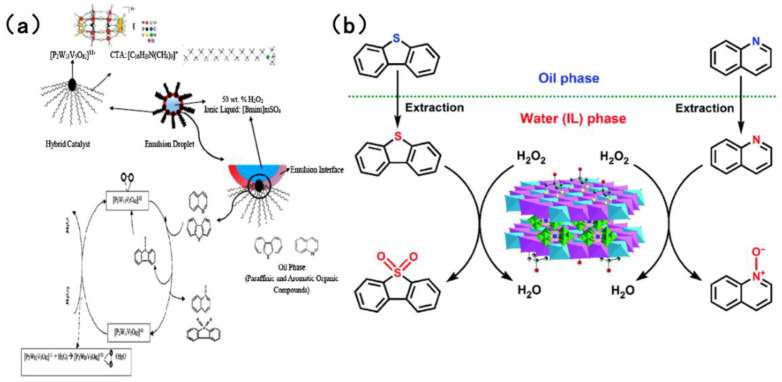
(**a**) Proposed mechanism of extractive-catalytic oxidative desulfurization and denitrogenation using ionic liquid. Reproduced with permission from Ref. [90]. Copyright 2017 Elsevier. (**b**) Proposed extraction and catalytic oxidative desulfurization and denitrogenation process by the catalytic system of H_2_O_2_/[bmim]BF4/TrisLDH-LaW_10_. Reproduced with permission from Ref. [91]. Copyright 2017 RSC.

In the field of catalysts for the simultaneous oxidation-based denitrogenation and desulfurization of organic compounds, polyoxometalate and heteropolyacid catalysts have attracted considerable interest [92]. The most prevalent of these are Keggin-type and Dawson-type polyoxometalate catalysts. These catalysts can be modified by incorporating transition metals such as vanadium to enhance their catalytic efficacy. As shown in Figure 11a, Banisharif et al. [90] developed an ECODS system that uses a vanadium-substituted Dawson-type emulsion catalyst alongside an ionic liquid (IL). Using H_2_O_2_ as the oxidant, this system efficiently removed dibenzothiophene (DBT) and quinoline from model fuels simultaneously. The long alkyl chains of cetyltrimethylammonium bromide (CTAB) in the emulsion catalyst were found to act as phase-transfer agents, facilitating the diffusion of DBT and quinoline from the non-polar oil phase into the polar solvent phase. Furthermore, the vanadium-substituted Dawson-type catalyst provided potent oxidation sites, activating H_2_O_2_ to generate reactive oxygen species. When in contact with -O-O- species on the catalyst surface, DBT is oxidized to DBTO_2_ and quinoline is oxidized to quinoline-N-oxide. Yao et al. [91] fabricated a Tris-modified layered double hydroxide (LDH) that was utilized as a support for the synthesis of a LaW_10_-type polyoxometalate (Tris-LDH-LaW_10_). Through electrostatic interactions and hydrogen bonding, the [LaW_10_O_36_]^9−^ active sites were immobilized within the LDH interlayers. Utilizing 30 wt% H_2_O_2_ as the oxidant, as demonstrated in Figure 11b, at 65 °C and atmospheric pressure, the ionic liquid facilitated the extraction of sulfur- and nitrogen-containing compounds from the oil phase to the aqueous phase. The [LaW_10_O_36_]^9−^ in the catalyst reacted with H_2_O_2_ to generate stable W-peroxide active species, oxidizing DBT to dibenzothiophene sulfones and quinoline to quinoline nitrogen oxides. Subsequently, the oxidation products were efficiently separated by the ionic liquid (via extraction), thus achieving simultaneous removal. Quinoline and DBT are both adsorbed on the active sites of the catalyst through π–π stacking and acid–base interactions. Quinoline has a slightly higher adsorption affinity than DBT, but due to sufficient active sites on the catalyst, the oxidation rate of DBT only decreases by less than 10%. Bertleff et al. [92] synthesized vanadium-substituted heteropolyacids with a Keggin structure. In high-temperature and high-pressure systems, molecular oxygen was selected as the oxidant. Conversely, in other systems, air was utilized as the oxidant. V^5+^ and Mo^6+^ in HPA-5 have multi electron redox ability. O_2_ reacts with the catalyst to generate reactive oxygen species (such as •OH). The introduction of vanadium enhances the oxidation selectivity of the catalyst for nitrogen compounds. Nitrogen compounds such as indole and quinoline first adsorb at the active site of the catalyst and are oxidized by the reactive oxygen species. Indole compounds generate carboxylic acids and CO_2_, while quinoline compounds generate quinoline N-oxides, which are further degraded into small molecule acids. The gaseous product is mainly N_2_. In both reaction systems, nitrogen compounds, excluding 1-methylindole, could be removed within an extremely short period. Furthermore, it was established that 1-methylindole, functioning as a reducing agent, effectively activated the catalyst, reduce part of V^5+^ in the catalyst to V^4+^ to form V^4+^/V^5+^ redox cycle and significantly enhancing the desulfurization efficiency across a range of simulated fuels.

**Figure 12:**
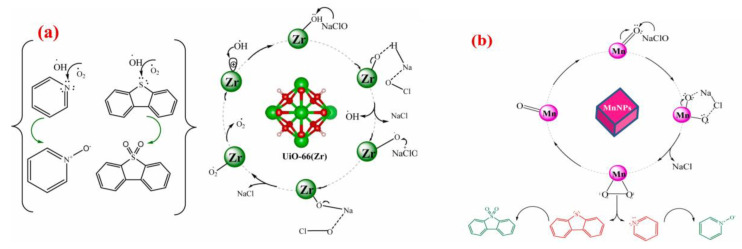
Proposed catalytic oxidation mechanism of Zr (**a**), and Mn (**b**) active species in MnO2/UiO-66 using NaClO as oxidant. Reproduced with permission from Ref. [98]. Copyright 2021 Elsevier.

Metal–organic frameworks (MOFs) are porous materials combining organic and inorganic characteristics [10]. They not only exhibit remarkable performance in adsorption but also demonstrate excellent capabilities in catalytic oxidation. MOFs provide adsorption sites for adsorption processes and mainly offer active sites in oxidation reactions, relying on active centers modified by metal ions or ligands [97,98] to activate oxidants and generate free radicals or reactive oxygen species. The previous section has reviewed various common MOF structures, and most of these structures can form derivatives via methods such as oxidation, pyrolysis, and coordination exchange [99], retaining some MOF characteristics or generating new structures. Palomeque et al. [93] selected ZrO_2_ (a UiO-66 derivative) as the support and tungstic acid (H_2_WO_4_) as the tungsten precursor, fabricating WO_x_/ZrO_2_ catalysts through two distinct methods: anion exchange (TA) and impregnation (AM), targeting the synergistic oxidative abatement of sulfur and nitrogen compounds in fuel oil. H_2_O_2_ forms the intermediate of hydroperoxytungstate, releases reactive oxygen species through peroxide bond breaking, and reacts with tetrahedral WO_4_^2−^ to generate W-O-O-H. Tungsten peroxide species attack the sulfur atom of sulfur compounds and the nitrogen atom of nitrogen compounds, and are oxidized to N-oxide sulfone and carboxylic acid products through nucleophilic attack. Due to the formation of hydroperoxytungstate intermediates, there is a problem of activity allocation caused by non-selective competition. Detailed characterizations revealed that the TA catalyst was dominated by tetrahedral tungsten species, which contributed to the formation of stronger Brønsted and Lewis acid sites—key active centers for ODS/ODN reactions. Consequently, it achieved a maximum desulfurization efficiency of 97% and denitrogenation efficiency of 95%, with superior reactivity toward refractory S/N compounds (e.g., sterically hindered thiophenes and pyridines). In contrast, the AM catalyst exhibited octahedral tungsten configurations, accompanied by reduced acid site density and the formation of crystalline WO_3_ phases. These structural features induced the agglomeration of tungsten species, weakened the metal-support interaction, and ultimately compromised catalytic performance compared to the TA catalyst. Density functional theory (DFT) calculations further elucidated the intrinsic activity mechanism of tetrahedral tungsten, verifying its lower reaction energy barrier for oxidant activation and pollutant cleavage. Subhan et al. [98] constructed a MnO_2_/UiO-66 composite catalyst by anchoring MnO_2_ nanoparticles onto UiO-66, employing NaClO as the oxidant for the simultaneous oxidative removal of DBT, indole, and other typical S/N contaminants. Experimental results demonstrated that the introduction of MnO_2_ nanoparticles significantly increased the specific surface area of UiO-66 by 88%, which facilitated the exposure of active sites (Mn^4+^ and Zr^4+^) and enhanced mass transfer during reactions. As depicted in Figure 12, the dissociation of NaClO produces ClO^−^, which interacts specifically with Mn^4+^ and Zr^4+^ active sites on the composite catalyst. This interaction triggers the heterolytic cleavage of ClO^−^, generating two highly reactive oxygen species with strong oxidizing capacity. These radicals selectively attack the sulfur atom in DBT and the nitrogen atom in pyridine, converting them into dibenzothiophene sulfone and pyridine N-oxide, respectively—both of which are easily separable from fuel oil. Notably, this catalytic system achieved complete (100%) removal of DBT and pyridine under ambient temperature and pressure, realizing rapid desulfurization and denitrogenation. Moreover, the by-products (NaCl and H_2_O) are inert and non-toxic, avoiding the introduction of impurities that could degrade fuel quality. Regarding the potential competitive relationship between sulfide and nitride removal, previous studies [100] have shown that nitrides such as pyridine and pyrrole have smaller sizes and adsorb into the micropores of the catalyst, preventing effective adsorption of thiophene and other compounds on the catalyst. By excessive use of oxidants, it had been demonstrated that sulfide removal is only affected by the competitive adsorption between the two, and the possibility of significant competitive oxidation has been ruled out. Therefore, the oxidation efficiency is highly dependent on the catalyst porosity.

**Figure 13:**
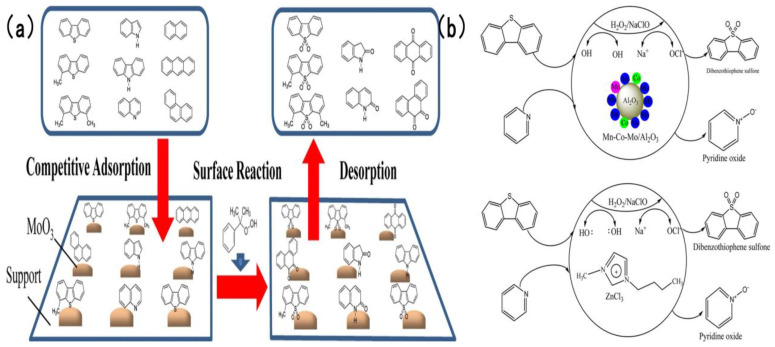
(**a**) Proposed ODS mechanism of dibenzothiophenes in the presence of nitrogen compounds and PAH. Reproduced with permission from Ref. [94]. Copyright 2020 Elsevier. (**b**) Proposed reaction mechanism for the CODS and CODN of DBT and pyridine in the presence of H_2_O_2_ and NaClO as oxidants over Mn–Co–Mo/Al_2_O_3_ and [Bmim]Cl/ZnCl2 as catalysts. Reproduced with permission from Ref. [95]. Copyright 2019 ACS.

In addition to the two types of catalysts discussed earlier, numerous studies have adopted supports including Al_2_O_3_ to fabricate highly efficient catalysts for simultaneous oxidative desulfurization and denitrogenation (ODS/ODN). Safa et al. [94] prepared a 12 wt% MoO_3_/γ-Al_2_O_3_ catalyst, using 80% cumene hydroperoxide as the oil-soluble oxidant, the active site of the catalyst adsorbs CHP and decomposed it into two strong oxidation species, t-Buo• and Mo^6+^-O-O-t-Bu and carried out an in-depth investigation into the mechanism underlying the influence of nitrogen compounds and polycyclic aromatic hydrocarbons (PAHs) on the oxidative removal of sulfur compounds. Experimental findings revealed that as the nitrogen concentration increased from 0 to 237 ppmw, the oxidation rates of 4,6-dimethyldibenzothiophene (4,6-DMDBT) and 4-methyldibenzothiophene (4-MDBT) declined remarkably as the content of impurities increased. Notably, 4-MDBT exhibited higher sensitivity toward impurities than 4,6-DMDBT. As illustrated in Figure 13a [94], gas chromatography-nitrogen chemiluminescence detection (GC-NCD) analysis validated that nitrogen compounds were partially oxidized during ODS. This suggests that nitrogen compounds compete against sulfur compounds for catalyst active sites and can be simultaneously removed. Subban et al. [95] developed a series of Al_2_O_3_-supported catalysts, encompassing Co-Mo/Al_2_O_3_, Ni-Mo/Al_2_O_3_, Mn-Co-Mo/Al_2_O_3_ and Mn-Ni-Mo/Al_2_O_3_. They evaluated and compared the oxidative removal efficiency of two oxidant systems: hydrogen peroxide (H_2_O_2_) and sodium hypochlorite (NaClO). Experimental results indicated that in the NaClO system at 25 °C, complete removal of dibenzothiophene (DBT) and pyridine was attained within 15 and 20 min, respectively—markedly superior to that in the H_2_O_2_ system. Figure 13b [95] depicts the detailed reaction mechanism: hypochlorite ions (ClO^−^) produced via NaClO dissociation possess stronger oxidizing power, integrated to the Mn active site and released after cleavage •OCl and enhanced coordination with Mn active sites than the peroxy species generated by H_2_O_2_, thus minimizing the unproductive consumption of the oxidant.

Most of the oxidants studied are mainly H_2_O_2_ or O_2_, while ozone, as a strong oxidant, has a very similar oxidation principle to other oxidants, which oxidizes organic sulfides to corresponding sulfones or sulfoxides. These oxidation products have enhanced polarity and are easily separated from the fuel by extraction, adsorption, or precipitation [101]. The difference is that O_3_ is oxidized without a catalyst. Ma et al. [101] used ozone generated by dielectric barrier discharge plasma combined with ionic liquid extraction to conduct deep oxidative desulfurization of simulated fuel. The order of oxidation activity of the four sulfides was TS > BT >> DBT > 4,6-dmdbt, which was different from that of the traditional hydrogen peroxide oxidation system. The [bmim] AC used has a stronger extraction ability for oxidation products, and the desulfurization rate of BT can reach 99.9%. Adhami et al. [102] used ozone microbubbles for oxidation to improve the contact efficiency between ozone and fuel and accelerate the oxidation reaction. The reaction is carried out through two paths: direct oxidation and free radical chain reaction. In the direct oxidation path, ozone molecule directly reacts with sulfide, breaks the C-S bond and gradually oxidizes the sulfur atom to sulfoxide (S=O, +4) and sulfones (O=S=O, +6). In the free radical chain reaction path, ozone first splits into •Roh, which grabs the hydrogen atom of sulfide to form R-S•, then the sulfur center radical reacts with ozone to form R-SO•, and finally the sulfoxide radical reacts with ozone or hydroxyl radical to form sulfone products. At present, there are few research papers on ozone denitrogenation or simultaneous denitrogenation and desulfurization, which is a challenging but promising field.

#### 4.3.3. Auxiliary Technology

To further enhance the performance of oxidative desulfurization and denitrogenation (ODS/ODN), integrating auxiliary approaches such as light irradiation or ultrasound into catalytic oxidation systems has notably boosted oxidation kinetics and process efficiency [96,103]. Photocatalysis operates by light excitation, prompting valence band electrons to undergo transition, leaving behind holes with strong oxidizing capacity; these can synergize with active sites generated by transition metals contained in the catalyst. Ultrasound-assisted technology employs ultrasound waves to generate physical effects that disrupt the aggregated state of sulfur and nitrogen compounds, alongside chemical effects that induce the cleavage of water molecules or oxygen to produce free radicals.

**Figure 14:**
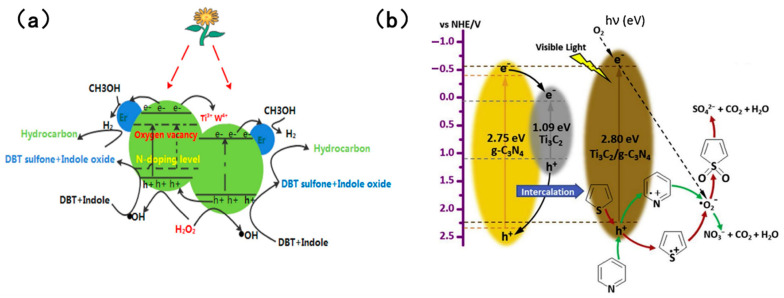
(**a**) Schematic presentation of charge carrier separation in the desulfurization and denitrogenation over Er/W-N-TiO_2_ aided by visible light. Reproduced with permission from Ref. [104]. Copyright 2021 Elsevier. (**b**) Proposed photocatalytic mechanism of PODN and PODS. Reproduced with permission from Ref. [86]. Copyright 2020 Elsevier.

A noteworthy study was reported by Aghbolagh et al. [96], who innovatively constructed a MAX-phase@rGO@PW_11_Zn composite photocatalyst to address the limitations of single-component catalysts, achieving efficient photocatalytic removal of sulfur and nitrogen compounds in fuel oil. When the catalyst dosage was 0.01 g and the reaction time was 60 min, the removal efficiencies of thiophene (TH) and pyridine (PY) reached 97.72% and 98.84%, respectively—markedly outperforming those of single-component catalysts. Wang et al. [104] fabricated an Er, W, N co-doped photocatalyst (Er/W-N-TiO_2_) and employed H_2_O_2_ as the oxidant for simultaneous denitrogenation and desulfurization. Figure 14a [104] illustrates the specific reaction mechanism: under visible light irradiation, valence band electrons of TiO_2_ are excited to the conduction band, generating photogenerated electrons and holes. Meanwhile, W^4+^/W^6+^ redox pairs, Er^3+^ ions, and oxygen vacancies in the catalyst effectively trap electrons, significantly suppressing electron–hole recombination. The holes oxidize H_2_O_2_ to produce hydroxyl radicals (•OH), which serve as the core active species for oxidizing sulfur and nitrogen compounds, while electrons are captured by methanol to generate hydrogen radicals (•H). Ultimately, sulfur compounds are oxidized by •OH into more polar sulfones, and nitrogen compounds are converted into nitrogen oxides, realizing deep purification of fuel oil. However, alkaline nitrogen oxides compete fiercely with sulfides for the active sites of the catalyst. Li et al. [79] developed a photocatalytic reaction system using Ti_3_C_2_/g-C_3_N_4_ as the catalyst and air as the oxygen source. Figure 14b [79] depicts the core mechanism: holes act as the primary active species driving desulfurization and denitrogenation reactions, preferentially undergoing electrophilic oxidation with pyridine and thiophene to form corresponding cationic intermediates (C_5_H_5_N^+^ and C_4_H_4_S^+^). In contrast, superoxide radicals ($•O_{2}^{-}$) generated by the combination of conduction band electrons with dissolved oxygen in air function as auxiliary active species, promoting further deep oxidation of the intermediates.

Eventually, pyridine is mineralized into NO_3_^−^, CO_2_, and H_2_O, while thiophene is converted into SO_4_^2−^ via sulfone intermediates.

Ultrasound assists oxidative desulfurization and denitrogenation (ODS/ODN) through its induced physical and chemical effects, featuring affordable economic cost and high removal efficiency for aromatic compounds. Notably, a review has elaborated on the key parameters in the auxiliary oxidation process, including temperature, ultrasonic power and frequency, pH value, initial concentrations of sulfur and nitrogen compounds, the role of phase transfer agents, extraction solvents, reaction kinetics, and fuel properties. Despite being a promising auxiliary technology, few studies have focused on simultaneous desulfurization and denitrogenation in recent years. Consequently, this technology remains largely at the theoretical stage, lacking sufficient in-depth experiments to be convincing.

In actual industrial production, sulfur oxides and nitrogen oxides are mostly removed by extraction [105]. Using the polarity difference between oxidation products and unreacted hydrocarbon fuels, sulfur oxides are extracted from non-polar fuels by selecting appropriate polar solvents [106]. Common traditional polar solvents include acetonitrile and methanol. Many studies [106] have shown that the solubility of oxidized organic sulfur compounds in acetonitrile is significantly increased, which can effectively separate sulfone products. In addition, ionic liquids have attracted extensive attention due to their designability, low vapor pressure and good extraction selectivity for sulfur/nitrogen compounds [54,107]. Ionic liquids can simultaneously or selectively extract sulfur and nitrogen compounds. Benzyl ionic liquids such as and have been proven to have high extraction desulfurization capacity for BT and DBT. Therefore, the combination of oxidation and extraction methods can achieve the goal of efficient removal of nitrides and sulfides, while avoiding secondary pollution. In addition, activated carbon, alumina, molecular sieve, MOF and other adsorption materials have been used for the removal of sulfur oxides [108] when the concentration of sulfur oxides is high or the selected oxidation system leads to a significant decrease in the solubility of the product. Oxidation products may form precipitates, which can be separated by physical methods such as filtration or centrifugation.

Simultaneous oxidative desulfurization and denitrogenation offers mild reaction conditions and high selectivity toward refractory sulfur and nitrogen compounds. It can also incorporate external auxiliary technologies like photocatalysis or ultrasound, enabling deep pollutant removal. However, challenges persist in practical applications: the commonly used H_2_O_2_ oxidant in reaction systems exhibits poor stability, catalyst costs are relatively high, and pollutants compete for catalytically active sites. Moreover, catalyst regeneration involves irreversible deactivation of active sites, making it difficult to fully restore performance, significant differences in regeneration mechanisms, and a lack of practical solutions. These issues result in a mismatch between oxidation methods and complex industrial systems, often remaining in the experimental stage.

### 4.4. Simultaneous Biodesulfurization-Biodenitrogenation

Biodesulfurization (BDS) and biodenitrogenation (BDN) utilize enzymes secreted by microorganisms to decompose sulfur compounds and nitrogen compounds in fuel oil. This technology boasts merits including low cost, minimal environmental pollution, and no adverse by-products. However, the slow growth rate of microorganisms and sluggish reaction kinetics necessitate prolonged degradation periods. In biocatalytic systems, attention must be paid to microbial selection, medium composition, reaction conditions, and the contact efficiency between microorganisms and substrates. Among the latest research advances, biofilms have been identified as a bridge facilitating metabolic and physiological interactions among microorganisms, and BDS-BDN systems based on biofilms are believed to surpass the limitations associated with suspended-cell BDS-BDN systems [2]. Currently, most studies have focused solely on either desulfurization or denitrogenation, with few reports documenting simultaneous biological desulfurization and denitrogenation. The most extensively studied pathway for BDS is the 4S pathway, which involves sulfoxidation, sulfonation, C-S bond cleavage to form sulfonates, and final oxidation to sulfates or sulfites. Beyond this, microorganisms can also achieve degradation via C-C bond cleavage: sulfur-containing compounds first undergo preliminary oxidation to weaken the stability of C-C bonds, followed by C-C bond cleavage catalyzed by specific enzymes. The fragmented carbon skeletons then enter the tricarboxylic acid (TCA) cycle of microorganisms and are ultimately mineralized into CO_2_ and H_2_O. The degradation mechanism of nitrogen compounds is strikingly similar to that of sulfur compounds; the key difference lies in the cleavage of C-N bonds instead of C-S bonds for nitrogen compound removal.

Dinamarca et al. [109] employed two bacterial strains, Rhodococcus and Cobetia sp. strain MM1IDA2H-1, to investigate the removal of dibenzothiophene (DBT) and quinoline (QN). In the mixed biofilm system, the shared carrier surface between the two bacterial strains results in reduced metabolic activity per unit area. Meanwhile, 2-hydroxybiphenyl—an intermediate product generated during DBT desulfurization by Rhodococcus—exerts a slight inhibitory effect on quinoline degradation. Consequently, the removal efficiencies of both DBT and QN by the mixed biofilm failed to exceed 80%. Similarly, Maass et al. [110] also utilized Rhodococcus for simultaneous denitrogenation and desulfurization of heavy gas oil (HGO). Notably, HGO concentration not only did not inhibit the growth of Rhodococcus but also significantly enhanced its growth rate at concentrations ranging from 10% to 50%. The overall trends of desulfurization and denitrogenation were consistent: the maximum removal efficiency was achieved at 40% HGO concentration, with desulfurization and denitrogenation efficiencies reaching 42.7% and 43.2%, respectively. Under the extreme condition of 100% HGO, severe cell aggregation occurred, accompanied by no obvious metabolic activity, and desulfurization/denitrogenation completely ceased. This verified that the deterioration of cell physiological status is the direct cause of the loss of catalytic activity. Subsequently, Maass et al. [111] conducted further investigations and identified the optimal culture conditions for Rhodococcus, achieving a 240% increase in biomass yield. However, experimental results showed that 10% HGO was the optimal concentration, which improved the denitrogenation efficiency by 8.3%. In contrast, the sulfur removal capacity was only 250 mg, significantly lower than the denitrogenation performance.

Simultaneous biological denitrogenation and desulfurization boasts core advantages such as mild reaction conditions and environmental benignity, making it well-suited for low-energy-consumption removal of sulfur and nitrogen compounds. Nevertheless, it suffers from limitations, including sluggish reaction rates and poor strain stability, leading to lower removal efficiency compared to technologies like hydrotreatment or oxidation. Despite these drawbacks, its unparalleled environmental friendliness endows biological methods with tremendous potential for future applications. With the development of various fields in biology, although various technologies have not been fully adapted to fuel desulfurization and denitrogenation, these other fields have demonstrated strong operability. For example, biofilm reactors [112,113] provide stable growth environments and efficient reaction interfaces for microorganisms, which can efficiently enrich microorganisms, enhance substrate mass transfer, and improve reaction stability. Metabolic engineering [114] aims to modify cellular metabolism and improve the degradation rate of sulfur and nitrogen compounds by optimizing microbial metabolic pathways. Synthetic biology [115] enables precise genetic modification of microorganisms to become efficient cells specifically designed for the removal of sulfides and nitrates.

## 5. Conclusions and Future Perspectives

Desulfurization and denitrogenation of fuel oil have long been a major challenge in industrial development. Over decades of exploration, most studies have centered on desulfurization, with its theoretical research relatively well-established. In contrast, research focusing on simultaneous desulfurization and denitrogenation remains extremely limited, and the mechanistic investigations into the co-removal of different pollutants are still incomplete. This review presents a comprehensive review and overview of hydroprocessing technologies and non-hydroprocessing technologies (adsorption, extraction, oxidation, and biological methods), highlighting the types of materials employed in each technology. It concludes each section with a summary and outlines the current challenges faced by each method or material, primarily including competition for adsorption sites or active sites between sulfur and nitrogen compounds. Furthermore, issues such as the high preparation cost of catalysts and their reusability have seriously hindered the industrialization process of various technologies. In addition, most of the research in the paper is on simulated fuel, which has certain differences in composition from real fuel. The aromatic hydrocarbons present in real fuels not only occupy the adsorption active sites, but also, due to their dense structure, easily form an adsorption layer on the catalyst surface, hindering the mass transfer of nitride and sulfide reactions. Meanwhile, the interaction between aromatic hydrocarbons and oxidation products such as sulfones delays product desorption, leading to blockage of catalyst pores. The most fatal aspect of simultaneous desulfurization and denitrogenation is the formation of stable coordination bonds between the nitrogen base and the active site, resulting in permanent deactivation of the active site.

Therefore, in the next 5 to 10 years, the industrialization process of non-hydrogen desulfurization and denitrogenation technologies for fuel will accelerate under the dual drive of the global ultra-low fuel standard “dual carbon” policy. The core path revolves around technological iteration, industrial adaptation, and ecological construction. Mainstream technologies represented by ODS/ODN, BDS/BDN, and ADS/ADN will break through the current bottlenecks of catalyst deactivation, high extractant cost, and slow degradation rate through the research and development of high-performance catalysts or extractants, and the optimization of reaction separation integrated processes. Among them, ODS and ODN need to focus on improving the balance between catalytic efficiency and oil yield, BDS/BDN relies on new reactors such as biological drip filtration towers to achieve continuous and automated operation, while ADS/ADN needs to enhance the cycling stability and large-scale preparation capability of MOF-based adsorbents. The following future research directions have been specifically proposed:

(1) Accelerate the transition of non-hydroprocessing technologies (e.g., adsorption, extraction, oxidation) from theoretical and experimental research to large-scale practical industrial applications. Reduce the fabrication costs of catalysts, extractants, and related materials, while developing catalysts/extractants with high activity, stability, and reusability.
(2) By making certain improvements, biotechnology that has been successfully applied in other fields can be adapted to the field of fuel desulfurization and denitrogenation. It is possible to cultivate highly efficient organisms in the fields of metabolic engineering and biosynthesis of solution fuels through techniques such as genetic modification.
(3) Integrate multiple technologies—for instance, coupling ultrasound-assisted oxidation with biological methods—to lower energy consumption while improving pollutant removal rates.
(4) Employ advanced in situ characterization techniques (e.g., STEM, FT-IR) to further unravel the specific processes and mechanisms underlying the competition for active sites and adsorption sites between sulfur and nitrogen compounds, laying a theoretical foundation for the design of high-performance catalysts and adsorbents.
(5) Most existing studies focus on simulated fuels, whose composition and component concentrations differ from those of real industrial fuels. Thus, the application of actual industrial fuels in experimental research is strongly advocated. Gradient processing technology is required for the treatment of oil products with high nitrogen and sulfur content, which involves first removing most of the target species through extraction and other techniques, and then using oxidation desulfurization and other methods to meet the standards.
(6) By adopting defect engineering and acid treatment or calcination strategies, controllable defects are constructed in catalytic materials such as MOFs and metal oxides, significantly improving catalytic activity and stability. Organic–inorganic hybrid phase transfer catalysts or molybdenum based composite materials should be designed to achieve synergistic oxidation conversion of sulfides and nitrides and deepen the reaction mechanism through multi metal active site synergistic design to solve the problem of decreased catalytic efficiency caused by competitive adsorption of sulfur and nitrogen compounds.

## Figures and Tables

**Table 2:** Multidimensional comparison of different oxidants.

Oxidant	Product	Environmental Risk	Industrial Costs	Operational Requirements	Oxidation Efficiency
H_2_O_2_	H_2_O, O_2_	No secondary pollution	low	Simple storage requirements; Easy experiment	higher
O_2_	oxide	Environmentally friendly	minimum	High pressure bottle storage; High voltage operation	high
TBHP	Methane, acetone, etc.	Serious pollution	high	High storage requirements; Complex experiment	highest
O_3_	O_2_, Oxidation by-products	Lower	high	Site preparation; High operational risk	lower

## Data Availability

Not applicable.
